# Determinants of health workers intention to use malaria rapid diagnostic test in Kintampo North Municipality, Ghana - a cross-sectional study

**DOI:** 10.1186/s12913-019-4324-6

**Published:** 2019-07-15

**Authors:** Michael Kurubire Anaba, Latifat Ibisomi, Seth Owusu-Agyei, Tobias Chirwa, Rohit Ramaswamy

**Affiliations:** 1 School of Public Health, Faculty of Health Sciences, University of the Witwatersrand, Johannesburg, South Africa; 2 Presbyterian Health Service, Agogo, Ghana; 3 Nigerian Institute of Medical Research, Yaba, Nigeria; 4 Kintampo Health Research Centre, Kintampo, Ghana; 5 The University of Health and Allied Sciences, Ho, Ghana; 6 Gillings School of Global Public Health, University of North Carolina, Chapel Hill, USA

**Keywords:** Intention to use, Rapid diagnostic test, Malaria, Kintampo North Municipality, Ghana

## Abstract

**Background:**

Resistance to antimalarial drugs resulting from overuse of the medication remains a threat to malaria control and elimination in endemic settings including Ghana. Reliance on clinical signs alone results in patients being diagnosed with malaria falsely. The World Health Organization and local guidelines recommend test-based diagnosis with malaria rapid diagnostic test (mRDT) or microscopy before prescription of antimalarial drugs. Despite the scale-up of mRDT through the procurement of mRDT kits and training of health workers on mRDT-led diagnosis of malaria, its use remains low with about 85% health workers reporting satisfaction with the presumptive diagnosis.

**Methods:**

A quantitative cross-sectional study was conducted to investigate the determinants of intention to use mRDT among health workers in Kintampo North Municipality, Ghana. A total of 110 health workers were surveyed from February to April 2017. Intention to use mRDT was measured as the primary outcome with a 5-item scale questionnaire based on the Technology Acceptance Model (TAM). We then tested its association with hypothesized determinants: coherence, cognitive participation, collective action, and reflexive action informed by the Normalization Process Theory (NPT) as well as health workers’ background characteristics using linear regression modeling.

**Results:**

The mean intention to use mRDT score was 82% (SD: 12.6). The regression model showed health workers intention to use mRDT was positively associated with coherence (β = 0.40, 95% CI 0.16–0.65) and cognitive participation (β = 0.36, 95% CI 0.15–0.58). Intention to use mRDT score was 6.85 units higher among health workers with three or more years of experience compared to those with less than 3 years of experience (β = 6.85 95% CI 0.59–13.12). However, intention to use mRDT score was inversely related to reflexive monitoring and collective action but not significant.

**Conclusion:**

The study identified that intention to use mRDT was positively influenced by health workers having a proper understanding of the aims and expected benefits (coherence) of the intervention and the availability of experienced staff and intervention champions (cognitive participation) to promote mRDT use among health workers.

## Background

Forty percent of the global population is at risk of malaria and the majority of this percentage resides in developing countries [1]. According to the World Health Organization (WHO), there were 212 million new cases of malaria in 2016 and Africa accounted for 90% of the recorded cases [2]. In Ghana, 2.3 million suspected cases of the disease were recorded at Out-Patient Departments (OPD) in the first quarter of 2017, which represent a 1.5% increase over cases reported during the same period in 2016 [3]. Early, accurate diagnosis with malaria rapid diagnostic test (mRDT) or microscopy and treatment with a recommended antimalarial drug is a key component of malaria control efforts [4]. However, resistance to antimalarial drugs resulting from overuse of the medication remains a threat to malaria control and elimination in endemic settings such as Ghana [5]. Reliance on clinical signs alone results in 32 to 93% (depending on the local malaria endemicity) of patients being diagnosed with malaria falsely and prescribed antimalarials [6–8]. The WHO recommends a test-based management strategy to ensure appropriate prescription of antimalarial drugs [4].

Ghana rolled out the test-based policy with mRDT-led diagnosis especially in rural facilities where microscopes are often unavailable in 2009 [9]. The national malaria control goal is to provide a correct diagnosis to all suspected malaria cases per treatment guidelines by 2020 [10]. To achieve this objective, there has been scale-up of mRDT through the procurement of mRDT kits and training of health workers on mRDT-led diagnosis of malaria [10]. Despite these efforts, mRDT use is still low [10]. Several studies have explored the sub-optimal implementation of mRDT both in Ghana [11, 12] and in other low and middle-income countries (LMICs) [13]. These studies have all examined health system factors affecting mRDT uptake. In the Rauf et al. study, 96% of Ghanaian health practitioners stated they knew about mRDT and expressed willingness to implement, yet 85% were satisfied with presumptive diagnosis [12]. Therefore, it is important to go beyond system factors and begin to consider agent factors. This is also reinforced by the multi-country study by Burchett et al. [13], which states that interventions to improve mRDT implementation were more successful when mRDT fit health practitioners priorities and therefore understanding the process by which actors engage with mRDT is a valuable extension to what is already known. This study responded to this gap through an investigation of factors that affects the intent of front-line staff to use mRDT in Kintampo North Municipality, Ghana.

### Conceptual framework

**Fig. 1:**
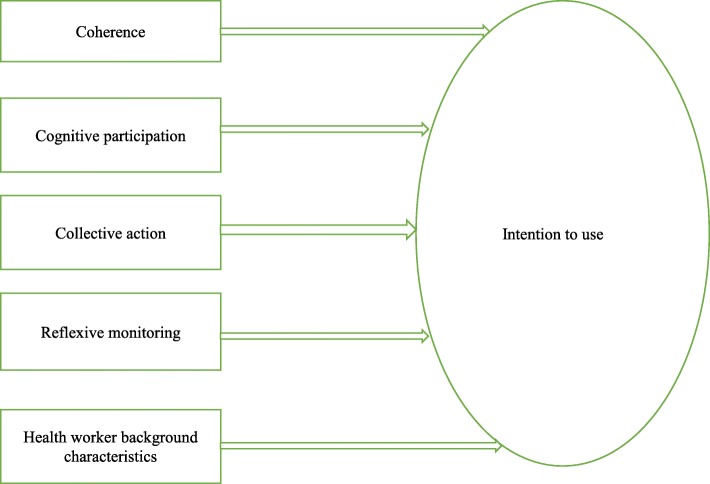
Conceptual Model on Malaria Rapid Diagnostic Test and its determinants among Health Workers. Adapted from NPT [16] and TAM [17]

The use of theory facilitates a better understanding of the generalisability and replicability of implementation interventions [14]. Despite this emphasis, it is recognized that there is no well-established method for theory selection or testing theories [14] and as a result, theories, models and frameworks have proliferated [15]. Therefore, theory selection needs to be governed by considerations about the fit of the theory to the research question [14]. To guide the research, a conceptual framework was adapted from two models [16, 17]. The adapted framework is depicted in Fig. 1. As stated above, the objective of this study was to look at actor specific factors, and a mid-range theory seemed most appropriate. Hence, the Normalization Process Theory (NPT) was selected as one of the theoretical frameworks.

NPT is a general-purpose mid-level theory, and the authors indicated that it can be adapted and used flexibly, which made it attractive for use in this study [18]. A recent systematic review [19] of NPT indicated that the theory was used extensively for process evaluation, and so there is a precedence of its use for the kind of research question of interest in this study. Examples are the use of NPT for evaluating provider-initiated testing and counseling in South Africa [20] and the surgical safety checklist [21]. Our model, therefore, assessed the constructs of NPT that were correlated with intention to use mRDT, since so many providers said they were willing but actual implementation was poor. The hypothesized determinants of intention to use were defined as follows: coherence (sense-making work done to enhance optimal use of mRDT), cognitive action (operational work done in order to successfully implement mRDT), collective action (the relational work done to build and sustain mRDT) and reflexive monitoring (appraisal work done to understand the effects of mRDT) [22]. Many of the technology adaptation models such as the Technology Acceptance Model (TAM) use intention to use as a proximal indicator for information technology use in healthcare [23], so we have used it as well. Studies have reported the effect of background characteristics of health workers on the use of health interventions [17, 24–26]. As such, we extended the NPT model to investigate further context-related predictors such as age, sex, the cadre of health worker and years of experience.

## Methods

### Study area

The study was conducted in health facilities across KNM municipality, Ghana. The municipality is in the middle belt of Ghana and covers an area of 7162 km2 with a resident population of approximately 134,970 [27]. The principal occupation in the area is farming followed by trading. The municipality was selected because of its high fever prevalence of 38% among children under 5 years, which is above the national prevalence of 19% [28]. Furthermore**,** malaria is the leading cause of under-five out-patient attendance in all the facilities in the area [27, 29]. The municipality has one public hospital, four registered private clinics, four health centers and 11 Community-based Health Planning and Services (CHPS) [30].

In the Ghanaian context, hospitals provide clinical care at the district level and serve an average population of 100,000 to 200,000 people [30]. They provide training and technical supervision to health centers at each district or municipality. They offer obstetrics and gynecology, child health, medicine, surgery services and are staffed with more skilled and competent health professional headed by a physician [30]. Health centers and clinics are often headed by a physician assistant and staffed with program heads in the areas of midwifery, laboratory services, public health, environment, and nutrition and serves a population of about 20,000 [30]. They provide basic curative and preventive services for adults and children, as well as reproductive health services and augment their service coverage with outreach services [30]. Basic preventive and curative services for minor ailments are being addressed at the community and household level by CHPS which are staffed by disease control officers, nurses, nurse assistants and community health officers (CHOs) [30].

### Study design

This was a cross-sectional study using a quantitative method.

### Study participants

The study participants included physician assistants, laboratory technologists, nurses and community health officers. These were the cadres of health workers that provide most health care in the primary health facilities in Ghana. Laboratory technologists typically hold a technician diploma or certificate in medical laboratory technology that enables them to collect and assist in processing, analysis and reporting of results of laboratory tests on specimens [30]. Community health officers possess a diploma in disease control or community health and perform follow up investigations of communicable and non-communicable diseases reported to the health facility [30]. Physician assistants hold an advanced diploma or bachelor’s degree in community medicine or physician assistantship and provide medical care in rural health facilities that lack physician [30]. Nurses may have a diploma or bachelor’s degree and provide nursing care in rural and urban facilities and prescribe medications in facilities that lack physicians or physician assistants [30].

The study was conducted in 19 of the 20 health facilities (one private health facility declined participation) in the municipality**.** Inclusion as a study participant for the survey required 6 months prior experience in the care of malaria patients. The health facilities provided a list of 125 staff that were directly involved in malaria case management and all were invited to participate in the survey. A total of 110 of the 125 health workers participated in the study.

### Data collection

The survey was in English and carried out from February to April 2017. The survey tool used was a structured interviewer-administered questionnaire. The first part of the questionnaire solicited for health workers’ background characteristics of age, sex, education, cadre of health workers, years of experience and geographic location. The second part asked questions that assessed the intention to use informed by the TAM. This was structured to elicit Likert scale responses ranging from 1-strongly disagree to 5-strongly agree. This tool has been empirically tested in several studies [31–34]. The third section included the four determinants of intention to use mRDT informed by the NPT NoMAD instrument [35]. These are coherence, collective action, cognitive participation and reflexive monitoring. The 5-point Likert scale was used here too. Four research assistants together with the corresponding author (who was the principal investigator for the study) collected the data.

### Quality assurance

The face validity and content validity of the draft questionnaire were assessed individually by three experts with extensive experience in the field of implementation science and malaria research as well as clinical experience in malaria care in Ghana. They were asked to comment individually on the face validity and content validity of the questionnaire and to judge whether the questions and possible answers were unequivocal. This resulted in minor modifications to the questionnaire. The survey tool was then pilot tested at a health facility in a neighbouring district-Kintampo South Municipality among 35 health workers to establish instrument reliability. Based on the results from this pilot study, the questionnaire was shortened and some questions were rephrased to enhance clarity. In ensuring data quality, research assistants were trained to enhance their understanding of the survey tool. The principal investigator regularly conducted on-site reviews. Data collected were checked for consistency and completeness before data entry.

### Data management and analysis

The collected data were coded, entered into Epi-Info version 7 and exported to STATA/SE Version 14 for analysis. Cronbach’s alpha was used to assess the internal consistency of intention to use the construct. The internal consistency of 0.72 for intention to use meant our scale provided a reliable and inter-related measure of the variable [36]. Thus, we computed a composite score for intention to use by summing the Likert scale scores of all the 5 questions that assessed the construct. Composite scores have been noted to increase measurement precision and limit the number of statistical tests needed in analyzing each of the constituent parts separately [35].

NPT to date has primarily been used as a qualitative research tool, but recently, the developers of NPT have created the NoMAD instrument [35]. Since the instrument itself is relatively new, the developers of NoMAD have provided some flexibility for researchers to demonstrate how best it should be used. The general recommendation is that for each item, the percentage of respondents who strongly agreed or agreed should be tallied and the more the positive rating for each construct, the more likely the intervention is likely to be normalized [35]. Summing the scores achieves the same effect since the larger the sum, the greater the positive rating of each construct. The developers of NoMAD and NPT have explicitly stated that this is a pragmatic measure and that they do not have a “formulaic process for scoring or combining items” [35]. Thus, we used an approach that makes the most sense to us given the high Cronbach’s alpha value showing high internal consistency of the variables. We computed composite scores by summing up all the item scores for each construct for linear regression analysis. Coherence included 4-items (a score of 4–20), collective action 4-items (a score of 4–20), cognitive participation 7-items (a score of 7–35) and reflexive monitoring 5-items (a score of 5–25). All the scores were converted to percentages for ease of use and interpretation.

For descriptive analysis, health workers background characteristics was operationalized as follows; sex (1-Female, 2-Male), cadre of health worker (1-Physician assistant, 2-Nurse, 3-Community health officer and 4-laboratory technologist), highest qualification (1-Bachelor’s degree, 2-Diploma, 3-Senior high certificate), years of experience and geographical location (1-Urban, 2-Rural). We reported these variables using frequencies and percentages while the intention to use, coherence, cognitive participation, collective action, reflexive monitoring and age were summarised with means and standard deviations.

## Results

### Study population

Table 1 summarizes the respondent characteristics. Of the 110 Health Workers, 57.3% were males. The mean age of respondents was 29.6 (SD: 5.8) years. The majority of the respondents were nurses (37.3%) and laboratory technologists were the minority (11.8%). Health workers with 3 or more years of experience were 52.7%. Those with a bachelor’s degree made up 13.6% of the sample while 63.6% were high school certificate holders. Two-thirds (66.4%) of the health workers were based in rural health facilities.

**Table 1:** Descriptive Statistics of the Variables used with Corresponding Mean Score of Intention to Use mRDT

Outcome Variable	Score Range (%)	Mean % (SD)	
Intention to Use	25–100	82 (12.6)	
NPT Constructs			
Coherence	45–100	78 (12.7)	
Cognitive participation	35–100	69 (12.8)	
Collective action	35–100	69 (12.8)	
Reflexive monitoring	40–95	72 (13.0)	
Background characteristic	*N* = 110 (%)	Mean Intention to Use % (SD)	*P*-value
Mean Age (SD)	29.6 (5.8)		0.07
Sex			0.06
Female	47 (42.7)	85 (10.8)	
Male	63 (57.3)	80 (13.5)	
The cadre of Health Workers			0.05
Physician Assistant	24 (21.8)	81 (11.6)	
Nurse	41 (37.3)	85 (10.3)	
Community Health Officer	32 (29.1)	81 (11.9)	
Laboratory Technologist	13 (11.8)	80 (16.7)	
Highest Qualification			0.58
Bachelor’s Degree	15 (13.6)	85 (14.7)	
Diploma	25 (22.7)	80 (11.5)	
High school Certificate	70 (63.6)	82 (12.5)	
Years of Experience			0.01*
< 3 years	52 (47.3)	82 (12.2)	
≥ 3 years	58 (52.7)	87 (9.9)	
Geographic Location			0.16
Urban	37 (33.6)	80 (12.3)	
Rural	73 (66.4)	83 (12.7)	

T-test for comparison of values between two groups, ANOVA for comparison of values between more than two groups

*SD* Standard Deviation, *N* Sample Size

* Statistically significant at *p* < 0.05

### Level of intention to use of mRDT among health workers

Table 1 also presents the scores of intentions to use mRDT by respondent characteristics. Among the health workers surveyed, the minimum intention to use score was 25% and the maximum score was 100% while the mean score was 82% (SD: 12.6). Health workers with less than 3 years of experience recorded the lower mean intention to use mRDT score (82%, SD: 12.2) compared to those with three or more years of experience (87%, SD: 9.9) and this differed significantly.

### Multiple linear regression model

Table 2 presents the coefficients of the (full) model fitted with all the determinants. The intention to use mRDT score increases by 0.40 for every unit increase in health workers’ understanding of the relevance and goals of diagnosing malaria with the innovation (coherence). The intention to use mRDT score also increased by 0.36 for every unit increase in health workers willingness to be involved in different aspects of the intervention implementation (cognitive participation). With regards to background characteristics, intention to use mRDT score was 6.85 units higher among health workers with three or more years of experience compared with respondents with less than 3 years of experience.

**Table 2:** Adjusted Coefficients of Determinants of Intention to Use mRDT

Determinant	Adjusted Coefficient (95% CI)
Coherence	0.40 (0.16, 0.65) *
Cognitive Participation	0.36 (0.15, 0.58) *
Collective Action	−0.20 (− 0.45, 0.04)
Reflexive Monitoring	− 0.04 (− 0.25, 0.16)
Age of health worker	0.43(−3.34, 4.21)
Experience of health worker	
Below 3 years	Ref
3 years and above	6.85 (0 .59, 13.12) *
Sex	
Female	Ref
Male	−1.71 (−6.34, 2.91)
The geographical location of health worker	
Urban	Ref
Rural	3.44 (−2.66, 9.55)
Highest qualification	
Bachelor’s degree	Ref
Diploma	1.29 (−5.84, 8.44)
High School Certificate	0.49 (−7.84, 8.84)
Cadre of health worker	
Physician assistants	Ref
Nurse	2.99 (−6.22, 12.20)
Community health officers	3.66 (−1.67, 9.00)
Laboratory technologists	1.07 (−5.72, 7.87)

*CI* Confidence Interval

∗*p* < 0.05

## Discussion

This study investigated the factors that affect the intention of health workers to use mRDT in the diagnosis of malaria in Kintampo North Municipality, Ghana. The study found an average of 82% intention to use score among the 110 respondents suggesting that the study population had an overall positive intention to use mRDT. The intention to use mRDT among health workers was influenced by clarity on the benefits and relative advantages (coherence) of mRDT. The finding resonates with the notion that the ability of intervention users to distinguish the relevance of a new intervention is a key determinant of implementation success [37, 38]. It is worth noting that in the era of presumptive treatment of malaria, case management guidelines required that all children under-five with fever in high-transmission settings including Ghana are prescribed antimalarial drugs [39]. This guideline might still have an impact on prescription behavior despite health workers awareness and willingness to use mRDT. Clearly communicating the goals of mRDT, advantages and how it differs from presumptive diagnosis of malaria is key to enhancing intention to use as a proximal indicator for mRDT use.

The intention to use mRDT among health workers in this study was enhanced by the availability of innovation champions or initiators and readiness for change practices (cognitive participation). This is consistent with a study in South Africa which found that using early intervention adopters or users of intervention as champions to drive implementation forward facilitated successful implementation of provider-initiated testing innovation [40]. The introduction of a new intervention to the clinical setting is often disruptive hence a comprehensive change management strategies involvement of staff, and the early identification of champions who are prepared to promote the initiative are important for optimal implementation [41]. Understanding front-line users’ needs and development of strategies to encourage inter-professional collaboration and protocols for new intervention use are crucial strategies for successful implementation of health technology like mRDT [42].

Evidence suggests that health workers are less likely to engage with innovation if they feel that its implementation had not eased their workload [43]. This validates our findings that availability of resources, enough time and staff (collective action) are negative predictors of intention to use mRDT among health workers in this study. There is evidence that suggests that health workers in urban health facilities are unable to fully implement the test-based management of malaria due to heavy clinic work-loads and that mRDT had altered health worker roles [44, 45]. Also, poor experience with the initial implementation of new interventions was more likely to result in implementation failure [40].

This study also found that having three or more years of experience was positively associated with mRDT compared to having fewer than three-year experience. This may indicate that the intention to use of mRDT comes from experience with mRDT use. These results are consistent with the report that having more years of experience is associated with higher health technology acceptance [46].

### Strengths and limitations

The study was conducted in only health facilities in KNM and this limits the generalizability of the finding beyond the study area. This was solely a quantitative study, and therefore qualitative approach may enhance a deeper understanding of context-specific factors that might influence intention to use mRDT. Lastly, this study was based on the intention to use as a proximal indicator of use, therefore we suggest future research should explore actual use of the innovation.

### Theoretical and practical implications

Using the NPT and TAM as theoretical guides in this study facilitated a deeper understanding of the process by which actors may engage in mRDT implementation which is critical for future research, practice and policy. This study has practical implications in particular for policymakers and healthcare professionals who strive to normalize mRDTs and similar technologies in developing countries like Ghana. Special attention should be paid to health care professionals who have less experience and those who show less intention of using mRDTs. Providing technical support may get them accustomed to new intervention which may boost acceptance and use [47]. Our study also underscores the importance of early adopter in motivating other health workers as well as showing the potential benefits of mRDTs. It has been found that when nursing staff thought the patient would benefit from new technology, the nursing staff themselves were more willing to actually use it. The opposite was also found when the anticipated benefits for the patient were thought to be low or unclear, thereby impeding the intervention use [47]. So, when introducing an evidence-based intervention like mRDT, health professionals must also believe in the benefits of the intervention.

## Conclusion

The study found that intention to use mRDT among health workers are influenced by clarity on the benefits and goals of the intervention and availability of innovation champions or initiators to promote the intervention use among health workers. Change management strategies such as technical support and training of frontline staff are also imperative in boosting the intention to use mRDT.

## Data Availability

The datasets used and/or analysed during the current study are available from the corresponding author on reasonable request.

## Abbreviations

CHPS: Community-Based Health Planning and Services
HREC: Human Research Ethics Committee
IMCI: Integrated Management of Childhood Illness
KNM: Kintampo North Municipality
MRDT: Malaria Rapid Diagnostic Test
NPT: Normalization Process Theory
TAM: Technology Acceptance Model
